# Beyond the Clock: Understanding Delays in Hip Fracture Surgery

**DOI:** 10.1177/21514593261424772

**Published:** 2026-03-06

**Authors:** Ella Davies, Benjamin H. L. Harris, Michael B. Fertleman, Louis J. Koizia

**Affiliations:** 1 Imperial College NHS Trust, London, UK; 2 Cutrale Perioperative and Ageing Group, Department of Bioengineering, 4615 Imperial College London, London, UK; 3 St. Catherine’s College, University of Oxford, Oxford, UK; 4 Department of Oncology, University of Oxford, Oxford, UK; 5 Institute for Liver and Digestive Health, Division of Medicine, University College London, London, UK; 6 Department of Psychosocial Rehabilitation, Medical University of Lodz, Lodz, Poland; 7 Collaborating Centre for Values-based Practice, St Catherine’s College, UK

**Keywords:** hip fracture, frailty, time to surgery, perioperative optimisation

The study by Akodu et al. examining the association between time to surgery and mortality following hip fracture repair in adults aged over 65 addresses a clinically important and enduring question in orthogeriatric care.^1^ The inclusion of a frailty index alongside traditional perioperative risk markers strengthens the analysis and reflects contemporary understanding of vulnerability in this population. However, caution is warranted in interpreting the observed associations, as delays to surgery in older adults frequently reflect necessary clinical stabilisation rather than just avoidable system inefficiency. Without accounting for the underlying reasons for delay, there is a risk of oversimplifying a complex care pathway and attributing causality where confounding by acute illness is also possible.

Older adults commonly present with hip fracture alongside acute medical conditions such as dehydration, infection, arrhythmias and delirium, many of which both precipitate the fall and independently increase perioperative risk. This broader vulnerability is reflected in population level data demonstrating strong associations between environmental stressors, physiological decompensation and hip fracture incidence, underscoring the complex clinical context in which these patients present. Infection and dehydration identified on admission, are well recognised contributors to postoperative delirium, which prolongs recovery, limits engagement and extends length of stay.^2,3^ Early identification and treatment of such conditions may improve outcomes but inevitably require time. Similarly, hypovolaemia is common following prolonged periods on the floor and must be corrected pre-operatively to reduce the risk of perioperative hypotension and acute kidney injury, complications strongly associated with mortality and prolonged admission in this population.^4^ These examples illustrate that some surgical delays are clinically appropriate and protective rather than detrimental.

Akodu et al. makes an important contribution to the ongoing debate on optimal timing of hip fracture surgery in older adults and reinforces the central role of timely operative management within orthogeriatric care. However, distinguishing clinically necessary optimisation from potentially avoidable organisational delay is essential to interpreting the observed associations and translating them into practice. Future analyses incorporating cause-specific categorisation of surgical and organisational delays would further strengthen this evidence base, allowing clinicians and health systems to pursue timely surgery while preserving the primacy of safe, individualised perioperative care for this complex and vulnerable population.
